# Altered EEG Signal Complexity Induced by Hand Proximity: A Multiscale Entropy Approach

**DOI:** 10.3389/fnins.2020.562132

**Published:** 2020-10-08

**Authors:** Philip Tseng, Yu-Hui Lo

**Affiliations:** ^1^Graduate Institute of Mind, Brain and Consciousness, Taipei Medical University, Taipei, Taiwan; ^2^Brain and Consciousness Research Center, TMU-Shuang Ho Hospital, New Taipei City, Taiwan; ^3^Psychiatric Research Center, Wan Fang Hospital, Taipei Medical University, Taipei, Taiwan

**Keywords:** entropy, multiscale entropy, complexity, EEG, action

## Abstract

Visual short-term memory (VSTM) is an important cognitive function that acts as a temporary storage for visual information. Previous studies have shown that VSTM capacity can be modulated by the location of one’s hands, where hand proximity enhances neural processing and memory of nearby visual stimuli. The present study used traditional event-related potentials (ERP) along with multiscale entropy (MSE) analysis to shed light on the neural mechanism(s) behind such near-hand effect. Participants’ electroencephalogram (EEG) data were recorded as they performed a VSTM task with their hands either proximal or distal to the display. ERP analysis showed altered memory processing in the 400–700 ms time window during memory retrieval period. Importantly, MSE analysis also showed significant EEG difference between hand proximal and distal conditions between scales 10 to 20, and such difference is clustered around the right parietal cortex – a region that is involved in VSTM processing and bimodal hand-eye integration. The implications of higher MSE time scale in the parietal cortex are discussed in the context of signal complexity and its possible relation to cognitive processing. To our knowledge, this study provides the first investigation using MSE to characterize the temporal characteristics and signal complexity behind the effect of hand proximity.

## Introduction

Visual short-term memory (VSTM) is an important cognitive function that acts as a temporary storage for visual information. Such storage allows visual and spatial information to stay intact and accessible in the brain although the actual physical stimulus is no longer in view (e.g., occlusion, blink, saccade, etc.). Such temporary information can then be accessed to support other functions such as goal-directed actions (e.g., Baddeley, 2002; Bridgeman and Tseng, 2011).

In the laboratory, VSTM integrity is often assessed with a change detection paradigm, which is similar to the popular spot-the-difference game, but in a more controlled laboratory setting. Participants see one image for a few 10 milliseconds, followed by a brief blank display, and then the image would reappear but sometimes may contain a slight change from its first appearance. The participant’s job is to respond whether the second image is totally identical to the first or not. Change detection tasks like these have been shown to positively correlate with one’s fluid intelligence (Kane and Engle, 2002; Fukuda et al., 2010). However, studies have also consistently shown that people’s VSTM performance is not as good as we subjectively think it is (Simons and Rensink, 2005; Simons and Chabris, 2011), and the capacity estimate on average is around 3 to 4 simple items (Bays and Husain, 2008; Luck and Vogel, 2013). Combining electroencephalogram (EEG) and event-related potentials (ERP) with a change detection task, Vogel and Machizawa (2004) have found that EEG signals near the right posterior parietal region showed larger amplitude as people’s VSTM load increased, suggesting an association between the right parietal cortex and VSTM.

Given the importance of VSTM and its link with various daily functions, the investigation of various methods to boost VSTM capacity, such as memory training (e.g., Shipstead et al., 2012; Blacker et al., 2014) and brain stimulation (e.g., Tseng et al., 2012b; Hsu et al., 2014), has attracted much attention in the field. Among these, one interesting yet under-investigated factor is the placement of one’s hands. That is, the closer the hands are to the things to be remembered, the better the memory is (for a review, see Reference Tseng et al., 2012a). This is known as the effect of hand proximity, or nearby-hand effect, and has been hypothesized to alter magnocellular processing (Gozli et al., 2012; Taylor et al., 2015) and attentional selection (Reed et al., 2006; Tseng et al., 2014). Specifically, this hand-proximity effect can enhance one’s change detection performance when hands are placed on both sides of the computer monitor, and this enhanced performance is most noticeable on the right side of the screen (Tseng and Bridgeman, 2011). The effect of hand proximity, however, is likely non-specific to VSTM since studies have also shown that placing one’s hands near the visual stimuli can facilitate attentional orienting (Reed et al., 2006; Sun and Thomas, 2013), slow down visual search (Abrams et al., 2008), speed up figure-ground segregation (Cosman and Vecera, 2010), shield attention from distraction (Davoli and Brockmole, 2012), and bias attention toward visual details (Davoli et al., 2012). These behavioral effects have been assumed to be the byproduct of bimodal neurons located in the premotor and parietal cortex (Reed et al., 2006), whose receptive fields follow the locations of the hands (Graziano and Botvinick, 2002). ERP evidence thus far has shown an amplitude increase that is non-selective (between target and distractors) during the early sensory stage (e.g., <200 ms from stimulus onset time), which then becomes target-selective in the later time window (e.g., >300 ms; Reed et al., 2013).

Although studies have begun to look into the electrophysiological basis of the effect of hand proximity, EEG studies remain scarce in the hand-proximity literature and no investigation has yet looked into the EEG effect of hand proximity beyond ERP. This is unfortunate because recent studies have shown that EEG, even in the absence of cognitive tasks, can deliver very promising results in neuroscience research and healthcare such as using EEG signals to classify between patients with Alzheimer’s disease, mild cognitive impairment (MCI; prodromal stage of Alzheimer’s disease), and healthy control (Mammone et al., 2019), as well as accurately predicting which MCI may eventually “convert” to Alzheimer’s disease in the future (Mammone et al., 2018). Therefore, to provide more insights to the electrophysiological signatures of the hand effect on VSTM, the present study aims to perform both the conventional ERP, as well as a multiscale entropy analysis (MSE), to quantify the possible different levels of complexity in EEG signals.

The MSE analysis is of importance here not only because of its novelty in the context of hand proximity studies, but it is able to better quantify and characterize the complexity and adaptability between two neural systems (i.e., hand proximal vs. hand distal in this case) than conventional ERP analyses. This is based on the assumption that biological systems need to operate across multiple spatial and temporal scales, and thus their complexity perhaps are also multi-scaled; and such information would not be observable in the traditional ERP approach. Indeed, in the medical field, MSE has already been applied to the analysis of EEG signals for two decades, and has been shown to be sensitive to the EEG differences between healthy and epileptic children (El Sayed Hussein Jomaa et al., 2019), healthy from MCI and Alzheimer’s disease patients (Morabito et al., 2012), and even between awake, light, and deep anesthesia (Li et al., 2010). In cognitive domains, previous research has also been able to use MSE to differentiate the EEG signals between good and poor performance in visuospatial working memory (Wang et al., 2014) and cognitive control (Liang et al., 2014; Huang et al., 2015), especially at higher time scales (e.g., 10–20 time scales). Notably, Wang et al. (2014) were able to show that, in the context of VSTM, physically active elderly adults performed better than inactive controls, and such behavioral distinction was also observable in MSE analysis at higher time scales. Therefore, if the effect of hand proximity is indeed acting on the adaptability and complexity of the system, we expect such modulatory effect to be visible in select time scales in the MSE analysis. This paper is organized as follows. Section 2 describes the VSTM task and how behavioral and EEG data were collected as well as preprocessed. Section 3 describes the behavioral and EEG findings, and contrasts the results from ERP and MSE approaches. Section 4 discusses the observed results and the potential usefulness of MSE in EEG studies, and the paper ends with a brief note on the theoretical implications of current findings in the literature of hand proximity.

## Materials and Methods

### Participants

Sixteen participants from the National Central University with normal or corrected-to-normal vision participated in this experiment. All participants gave informed consent prior to their participation. Two participants were excluded from analysis due to too many movement artifacts in their EEG data, which resulted in eight male and six female subjects (mean age = 23) taken for the analysis. All experimental procedures were approved by the Institutional Review Board of National Cheng Kung University Hospital.

### Task and Procedures

**FIGURE 1:**
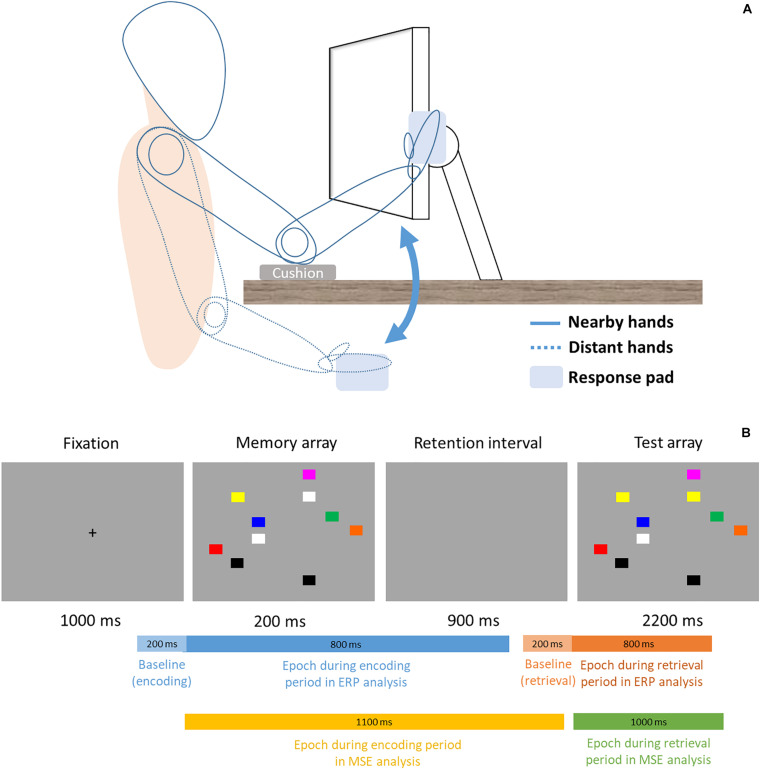
**(A)** Setup and Posture. Participants sat 48 cm in front of the computer display. In the proximal block (solid lines), participants rested their elbows on the desk and placed their palms sideways next to the monitor, where a response pad is attached and slightly titled/rotated to fit the orientation of the fingers better. In the distal block (dotted lines), participants placed their hands and the response pad on the lap. **(B)** Trial procedure and time ranges of epochs in ERP and MSE analyses. For ERP, a 200 ms epoch (light blue and light orange) is used to serve as baseline for the signals that come after. Note the bars are not drawn to scale.

This study used a within-subject design, thus the formal experimental session was divided into two blocks (proximal vs. distal) in counterbalanced order across all participants. Participants’ sat 48 cm from the monitor and, in the proximal session, placed both hands right next to the monitor with cushion below their elbows (Figure 1, solid lines). In the distal session, participants’ hands were placed on their lap under the desk (Figure 1, dotted lines). In each session, participant performed a change detection task similar to the Tseng and Bridgeman study (2011, Experiment 2). The task consisted of 144 trials. In each trial, participant was instructed to memorize an array of 10 colored rectangles (16 × 13 mm), and compare it with a subsequent display to indicate whether any one rectangle had changed color. This would be analogous to the experimental and computerized version of the “spot-the-difference” game, except that we used simple color squares and that the two displays were presented in succession. The locations of the squares were randomized across every trial. Unlike the original Tseng and Bridgeman (2011) study that used colors of contrasting brightness, the present study used eight similarly darker colors (red, yellow, green, dark cyan, orange, blue, purple, gray) to control for the varying degrees of brightness that was present in the original study. Half of trials contained a color change of one square and the other half did not. Each trial began with a 1000 ms fixation cross, then sequentially followed by a 200 ms memory array, 900 ms retention interval, and a 2200 ms test array. The entire trial would be over with one sequence (i.e., one-shot change detection) in a total of 4300 ms per trial. There was no repetition of the memory or test arrays, nor could the participants switch to previous displays and look again. During the test array presentation time (2200 ms), participants simply had to judge whether a color change was present or not by pressing one key for “change” and another for “no change” with their right index and middle fingers (all participants were right-hand dominant). Participants’ EEG signals were recorded concurrently as they performed the change detection task.

This one-shot change detection task is designed in a way that participants are primarily concerned with encoding information on the display during the memory array, and retrieving such stored information for comparison during the test array, without too much overlap between the two stages. Because of this well-segregated temporal structure, cognitive processes or neuroimaging signals during these two distinct time windows have often been referred to as the encoding period (i.e., memory array) and retrieval (i.e., test array) period. Accordingly, the present study also uses the same structure to segment the event-related EEG signals.

Participants’ VSTM capacity was estimated using Cowan’s *K*, that is computed from the VSTM memory array set size (S), which is 10 in this study, and participants’ hit rate, or true positive rate (TPR), and false alarm rate, or false positive rate (FPR) (Cowan, 2001):

(1) K=S×(TPR-FPR)

The hit rate, or TPR, was defined as the conditional probability that the participants responded “change-present” when a color change indeed took place. The false-alarm rate, or FPR, was defined as the conditional probability that the participants responded “change-present” when there was in fact no changes in color. The difference between TPR and FPR is then multiplied by S, which is the set size, or memory load, of the VSTM stimuli (i.e., 10). We conducted a paired *t*-test to test whether hand proximity would improve participants’ VSTM performance by comparing the mean *K* values between the proximal and distal conditions.

To test for any left or right bias that was previously reported in the literature (Tseng and Bridgeman, 2011), where proximal hands were found to induce a bias toward the right side of the screen in right-handed participants (Le Bigot and Grosjean, 2012), participants’ regional gain (or bias, *B*) was also computed relative to their hit rates from the distal condition:

(2) $$B=\frac{\mathrm{Proximal}\,\mathrm{true}\,\mathrm{positive}\,\mathrm{rate}}{\mathrm{Distal}\,\mathrm{true}\,\mathrm{positive}\,\mathrm{rate}}$$

The display would be divided into the left, center, and right region, and *B* would computed for each region to give a proportional estimate of the hand-driven bias toward a certain region over and above the distal baseline. The transformation of hit rates into *B* ratio is needed because the center region is always the region with highest hit rates, but such hit rates would only reflect participants’ natural tendency to look at the middle of the screen, and mask the lateral bias that may be introduced by hand proximity. Therefore, a ratio that takes the original hit rate from the distal baseline is more suitable to reveal such a directional shift of attentional focus.

### Electroencephalography Recordings

Electroencephalogram activity was recorded with Ag/AgCl electrodes mounted in an elastic cap using a 32-electrode arrangement following the International 10–20 System, referenced to the left and right mastoid. Vertical and horizontal electro-oculograms were also recorded. Electrode impedances were kept below 10 kΩ for all electrodes. The online low-pass filters were set at 300 Hz. Data were recorded with Neuroscan software, with a sampling rate of 1000 Hz.

### Event-Related Potential Data Analysis and Averaging

The continuous EEG data was applied a digital low-pass filter of 30 Hz (24 dB/octave) in order to filter out high-frequency noise. The EEG data were then segmented into epochs that starts from 200 ms before the (memory or test) array onset, and continues until 800 ms after the same array onset. Baseline correction was executed using a pre-stimulus interval by subtracting averaged pre-stimulus voltage from each EEG data point in the whole epoch. Epochs with artifacts fluctuating over ±100 μV and incorrect response were rejected. Each trial was divided into two segmented epochs including encoding (i.e., memory array display) and retrieval (i.e., test array display) waveforms. ERP analysis was performed by averaging artifact-free trials based on stimulus type (i.e., proximal vs. distal conditions). In the encoding period, all artifact-free trials were averaged based on proximal and distal conditions. In the retrieval period, only true-positive trials were averaged in proximal and distal conditions.

In order to investigate the neurophysiological mechanism of the hand proximity, a three-way repeated-measure ANOVA with the factors of within-subject factors of hand proximity (proximal vs. distal), anterior/posterior electrodes (frontal vs. central vs. parietal regions), and laterality (left vs. middle vs. right) was conducted based on the mean amplitude from 400 to 700 ms after onset of memory or test array in both proximal and distal sessions. Three scalp regions were chosen to perform the statistical analysis as a within-subject factor of anterior/posterior electrodes: frontal (F3, FZ, F4), central (C3, CZ, C4), and parietal regions (P3, PZ, P4). Another within-subject factor was laterality: left (F3, C3, P3), middle (FZ, CZ, PZ), and right (F4, C4, P4). The other within-subject factor was hand proximity (proximal vs. distal conditions). Greenhouse–Geisser correction was applied to repeated measures with more than one degree of freedom.

### MSE Analysis

Complexity in EEG signals at different time scales was analyzed with MSE analysis (Costa et al., 2002, 2005; Goldberger et al., 2002). The electrode of interest here is P4, since activities in the posterior right parietal cortex have been repeatedly shown to be critically involved in the visuospatial change detection task employed here (Tseng et al., 2010, 2013; Juan et al., 2017). MSE analysis was performed from time scale 1 through 25 both for the encoding/retention stage (0–200 ms in the memory array through 0–900 ms in the retention interval) and the retrieval stage (0–1000 ms in the test array) of the change detection task (Figure 1B). This was done in two steps: first, the algorithm down-samples the EEG post stimulus time series {x*_1_*,…, x*_*i*_*,…, x*_*N*_*} for every trial in each condition. The down-sampling procedure used a coarse-grained procedure along different time scales: for timescale τ, the coarse-grained time series was obtained by averaging data points within non-overlapping windows of length τ. Thus, each element of a coarse-grained time series, denoted as *j*, is calculated as:

(3) $$y_{j}^{\left(\tau \right)}=\frac{1}{\tau }+\sum_{i=\left(j-1\right)\,\tau +1}^{j\,\tau }x_{i},1\leq j\leq \frac{N}{\tau }$$

We then compute the sample entropy for each coarse-grained time series. Sample entropy is defined by the negative natural logarithm of the conditional probability that a time series of length *N*/τ, having repeated itself within a tolerance *r* (similarity factor) for *m* points (pattern length), will also repeat itself for *m* + 1 points, without allowing self-matches. Note that the tolerance factor *r* is set as the percentage of the signal SD, and it is calculated for scale 1, then kept fixed for all the other scales.

Due to the scarcity of MSE studies in human EEG signals, there is no golden standard or consensus on the best parameters for calculating sample entropy. However, some studies using clinical applications have suggested the parameters of *m* = 1 or 2 and *r* = 0.1 to 0.25 to provide a high validity for sample entropy in EEG signals (e.g., Escudero et al., 2006; Takahashi et al., 2009; Yang et al., 2013). With these suggested parameters we have also obtained good results in the past when analyzing EEG signals in the context of cognitive tasks similar to the current study (Wang et al., 2014; Huang et al., 2015). Specifically, in this study the pattern length, *m*, was set to 1 (i.e., one data point was used for pattern matching). The similarity criterion, *r*, was set to 0.30, meaning that data points were considered to be indistinguishable if the absolute amplitude difference between them was ≤30% of the time series standard deviation (Huang et al., 2015). Data preprocessing was performed using SPM8 (Statistical Parametric Mapping) and custom MATLAB (MathWorks) scripts^1^. Paired *t*-tests were conducted, scale by scale, to test the difference of sample entropy between proximal and distal conditions among 32 channels, including Fp1, Fp2, F7, F3, Fz, F4, F8, FT7, FC3, FCz, FC4, FT8, T3, C3, Cz, C4, T4, TP7, CP3, CPz, CP4, TP8, A1, T5, P3, Pz, P4, T6, A2, O1, Oz, O2. Due to the number of paired *t*-tests we are performing, we adjusted *p*-value for multiple comparisons by taking into account the false discovery rate (Tseng et al., 2010), with significance level set at *p* < 0.05.

## Results

### Behavioral Results

**FIGURE 2:**
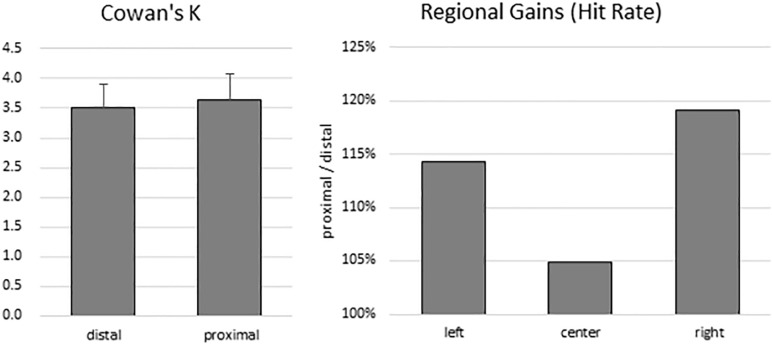
Behavioral results. There was no difference in VSTM capacity estimates between the distal and proximal conditions (left panel). However, despite the absence of enhanced change detection performance with hand proximity, participants’ attention was still biased to the right side of the display (right panel).

To estimate participants’ VSTM capacity, Cowan’s *K* was computed for both proximal (mean: 3.63; range: 0.69–6.81) and distal (mean: 3.51; range: 0.97–5.83) conditions. There was no significant difference between *K* values between the distal and proximal conditions (*t*(13) = 0.519, *p* = 0.612) (Figure 2, left panel). Because the sample size was small, a non-parametric test was also conducted. Wilcoxon signed-rank test showed that the effect of proximity did not elicit a statistically significant (*Z* = −0.668, *p* = 0.504) difference. Indeed, median *K* values of proximal and distal conditions were 3.40 and 3.47, respectively.

To test for any lingering traces of the effect of hand proximity, we explored whether a difference in regional gains may persist. As in the Tseng and Bridgeman (2011) study, we broke down the hit rates into left, center, and right regions according to the location of change for both the proximal and the distal conditions. We then divided the proximal hit rates from the distal hit rates, which gives a proportional estimate of the hand-driven bias in relation to the distal baseline (Figure 2, right panel). These trends do show a stronger right bias (almost 20% more than the distal condition), mild left bias, and a weak center enhancement in terms of hit rates, though they do not show a statistically significant difference in a one-way repeated-measures ANOVA (between left, center, and right: *F*(2,26) = 0.360, *p* = 0.621). These trends are consistent with the observations from the Tseng and Bridgeman (2011) study, which in the absence of statistical tests also showed a rightward bias in the proximal condition.

### EEG Results

#### Encoding Period

**FIGURE 3:**
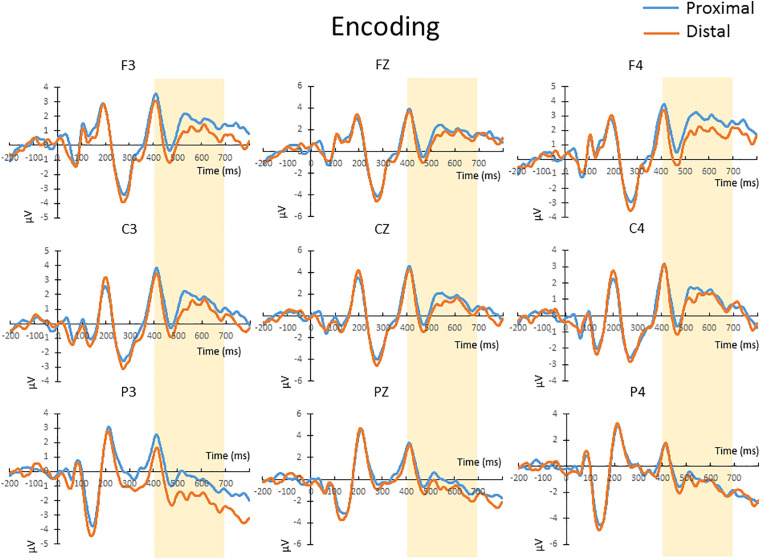
The waveforms in the proximal (blue) and distal (orange) conditions during the encoding period. There was a marginally significant (*p* = 0.054) difference between the proximal and distal conditions, which is likely a result of a higher overall activity in the proximal condition (blue). No further *post hoc* tests were performed, since there was no significant interaction between hand proximity and other factors.

The mean amplitude from 400 to 700 ms after the onset of memory array, which covers the time window implicated for memory encoding/maintenance from previous studies (Jolicoeur et al., 2008; Tseng et al., 2012b), were submitted to a repeated-measures 2 × 3 × 3 ANOVA. There was no significant main effect of hand proximity [*F*(1,13) = 4.487, *p* = 0.054], or any significant interaction between hand proximity and other factors, including anterior/posterior electrodes [*F*(2,26) = 0.291, *p* = 0.663], laterality [*F*(2,26) = 0.630, *p* = 0.459], or three-way interaction among all factors [*F*(4,52) = 1.283, *p* = 0.295]. These results suggest a marginally significant effect of hand proximity, where activities from the proximal condition seem to be higher across several channels (Figure 3, Channels F3, C3, and P3).

**FIGURE 4:**
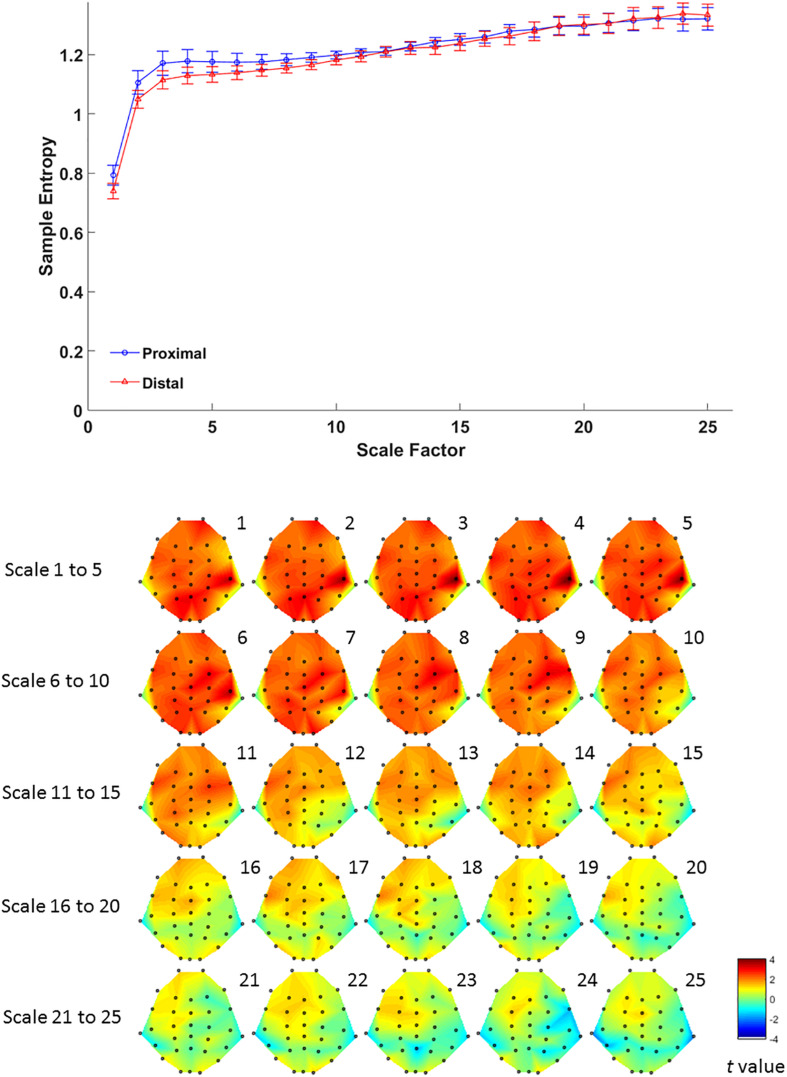
Upper panel: difference in EEG-based multiscale entropy (*m* = 1, *r* = 0.3) at P4 electrode in the proximal (blue) and distal (red) conditions during the encoding period in all trials. Lower panel: contrast of MSE between proximal and distal conditions among 32 channels. Colors represent *t*-values from paired-samples *t*-test between the proximal and distal conditions.

For complexity, MSE analysis showed no MSE difference between proximal and distal conditions. Although there seemed to be a trend in lower time scales (Figure 4, lower panel), such trend did not reach statistical significance after FDR correction for all time scales (all *p* > 0.05 FDR corrected, as shown in Figure 4). This suggests that hand proximity may have similar effects on brain signal complexity during encoding period.

#### Retrieval Period

**FIGURE 5:**
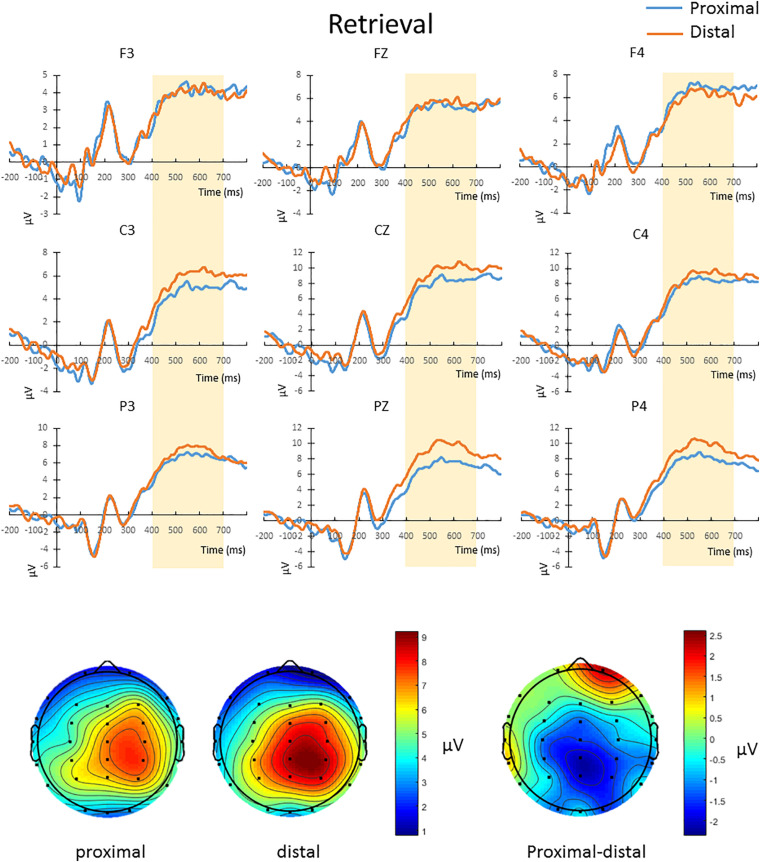
Upper panel: The waveforms in the proximal (blue) and distal (orange) conditions during the retrieval period in all hit trials (i.e., correctly detecting a color change). There was a three-way interaction driven by the effect of hand proximity that was only significant at the right posterior sites (Pz and P4, bottom row). Note that the proximal condition actually has lower ERP amplitude and peak than the distal condition, supporting the idea of a magnocellular and hand-induced impairment that occurs specifically during the retrieval period. Bottom panel: The lower ERP amplitude introduced by hand proximity seems to be driven by the right parietal region, which is depicted on the right as a contrast between proximal and distal conditions, though we note that results at the electrode level should be interpreted with caution.

The mean amplitude from 400 to 700 ms after the onset of test array in correct change-detection trial conducted a repeated-measures 2 × 3 × 3 ANOVA. The main effect of hand proximity was not significant [*F*(1,13) = 0.276, *p* = 0.608]. There was also no significant interaction between hand proximity and laterality [*F*(2,26) = 2.565, *p* = 0.097]. However, we observed a significant interaction between hand proximity and anterior/posterior electrodes [*F*(2,26) = 6.877, *p* = 0.015], as well as a significant three-way interaction [*F*(4,52) = 6.069, *p* = 0.005]. To further explore the three-way interaction, we first conducted a two-way ANOVA with hand proximity and laterality as within-subject factors in frontal, central and parietal regions. The interactions between hand proximity and laterality in these regions were not significant [frontal: *F*(2,26) = 0.767, *p* = 0.469; central: *F*(2,26) = 0.580, *p* = 0.497, parietal: *F*(2,26) = 2.994, *p* = 0.088]. However, the main effect of hand proximity was statistically significant only in the posterior and parietal regions [frontal: *F*(1,13) = 0.004, *p* = 0.950; central: *F*(1,13) = 3.889, *p* = 0.070, parietal: *F*(1,13) = 4.846, *p* = 0.046]. Separate comparisons showed that, within the parietal regions, the effect of hand proximity is only significant at Pz [*t*(13) = −3.568, *p* = 0.003] and P4 [*t*(13) = −2.576, *p* = 0.023], but not P3 [*t*(13) = −0.683, *p* = 0.507], suggesting a right parietal involvement in hand proximity (Figure 5, channels Pz and P4). To our surprise, however, the significant differences at Pz and P4 are driven by a lower overall and peak amplitude in the proximal condition, instead of the other way around as one might suspect.

**FIGURE 6:**
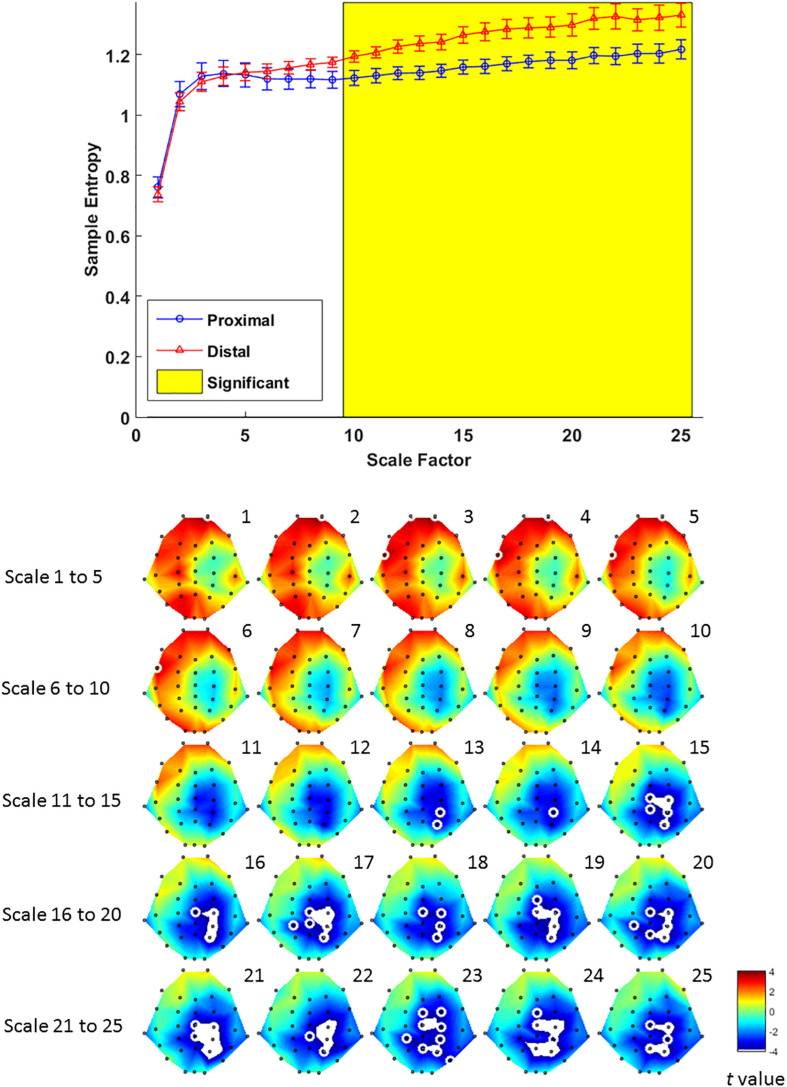
Upper panel: difference in EEG-based multiscale entropy (*m* = 1, *r* = 0.3) at P4 electrode in the proximal (blue) and distal (red) conditions during the retrieval period in all hit trials (i.e., correctly detecting a color change). The yellow region denotes significant different between proximal and distal conditions (*p* < 0.05, FDR corrected). Lower panel: contrast of MSE between proximal and distal conditions among 32 channels. Colors represent *t*-values from paired-samples *t*-test between the proximal and distal conditions. For each scale, the EEG electrodes enclosed by white circles denote that the difference of sample entropy between proximal and distal conditions on these electrodes was significant (*p* < 0.05, FDR corrected)(Due to the number of channels and scales available, it is possible that not all channels/scales are normally distributed. Thus a cluster-based non-parametric permutation (CBnPP) test (Maris and Oostenveld, 2007; Groppe et al., 2011) was also conducted to test the differences of multi-channel MSE between two conditions during the retrieval stage. The contrast of MSE between proximal and distal conditions among 32 channels in CBnPP test is similar to the contrast in paired *t*-test with *p* < 0.05 false discovery rate correction. The non-parametric test revealed the same results as Figure 6, where complexity between proximal and distal conditions diverged significantly from scale 10 and on at P4. In terms of topography, the non-parametric test revealed more significant channels, but also in the central and parietal regions as Figure 6. Because of the high degree of similarity between the parametric and non-parametric tests, and that the parametric tests seemed to be more conservative with fewer electrodes with FDR, we have kept the results from the parametric tests in the main “Results” section).

Multiscale entropy results showed that signal complexity from time scales 13–25 in proximal condition was significantly lower than its counterpart from the distal condition, over EEG channels from mid-central to right parietal brain regions (Figure 6, lower panel). The effect of hand proximity was significant within right parietal regions. Therefore, we compared the effect of hand proximity on MSE at P4 electrode. For scales from 10 to 25, the MSE at P4 was higher in distal condition than proximal condition during retrieval period (Figure 6, upper panel). These MSE results were consistent with our ERP findings, though it should be noted that these are electrode-level findings, and thus localizations at P4 location should be interpreted cautiously.

## Discussion

The present study aimed to test whether hand proximity would alter neural processing at varying levels of complexity. To this end, we observed that at scale 10 and beyond, EEG signal complexity becomes significantly different between the hand proximal and distal conditions. To our knowledge, this is the first evidence documenting altered visual processing near the hands based on entropy and MSE analysis. This fills the void of traditional ERP analyses, since such average-based analysis cannot provide enough insight regarding differences in the low-frequency range at higher MSE time scales.

### Effect of Hand Proximity on Neural Processing: Location and Timing

The findings from EEG data are twofold: location and timing. In terms of location, both EEG analyses suggest activities in the parietal region to be responsible for the effect of hand proximity. Although the right parietal cortex has long been hypothesized to be involved in the effect of hand proximity (e.g., Reed et al., 2006; Tseng et al., 2012a), the present study is able to provide electrophysiological evidence for the sensor locations underlying the near-hand effect. In ERP results, a significant difference between distal and proximal condition is only observed in the parietal sites (Figure 5). MSE analysis is perhaps more specific, and shows that such altered neural processing in terms of signal complexity is also concentrated in the right parietal region (Figure 6, lower panel). Therefore, both analyses suggest a parietal involvement behind the effect of hand proximity on visual processing. This can perhaps be linked to the bimodal neuron account originally put forth by Reed et al. (2006), who proposed that the bimodal neurons whose receptive field move along the egocentric hand-centered coordinates (Graziano and Botvinick, 2002) may be a contributing factor for the effect of hand proximity on visual processing.

In terms of timing, there are two levels of temporal characteristics worth discussing: one at the cognitive stage level (encoding vs. retrieval stage), and the timing of EEG signals within a particular cognitive stage. At the cognitive stage level, both MSE and ERP results showed a pronounced proximal-distal difference in the retrieval period (Figures 5, 6), and less so in the encoding period (Figures 3, 4). Therefore, although hand positions are fixed throughout the entire trial and block (i.e., participants hands were near the display in both encoding and retrieval stages), the effect of hand proximity on EEG signals is not constant at every stage of cognitive processing that mediate VSTM. This suggests that hand proximity is not a simple additive factor to whatever cognitive process that is being carried out at the moment; rather, it interacts with the task (and its associated cognitive demand) at hand. Although counter-intuitive, this observation is actually consistent with previous neuropsychological (Berryhill and Olson, 2008) and EEG (Hsu et al., 2014) studies that suggest an important role for the parietal cortex in VSTM retrieval. Specifically, boosting parietal activities prior to the experiment with external stimulation also alters parietal activities throughout the experimental session but mostly at the VSTM retrieval stage (Juan et al., 2017). In a similar vein, Reed et al. (2013) used a target detection task combined with hand proximity and found an alteration to EEG signals that is non-selective in the sensory window, and selective for task-specific targets in the later time window. Indeed, our previous MSE study comparing EEG signals between physically fit and unfit elderly adults while the participants performed a VSTM task also showed marked complexity differences in the memory retrieval period, but not the encoding period (Wang et al., 2014). Therefore, the attentional effect of hand proximity is not uniform at every stage of the task although hand positions were kept in place throughout the entire block. In this light, our findings here converge on the same conclusion, and suggest that hand proximity induces a task-dependent modulation of attentional processes during the memory-retrieval stage of VSTM processing.

Regarding finer-level temporal characteristics of EEG signals within the retrieval stage, we observed that the effect of hand proximity on neuronal processing is more evident at a later time window. In other words, we did not observe a change in early sensory components (e.g., N1, P1). This is first evident in the ERP analysis, where the distal-proximal difference is observable in the 400–700 ms window during the retrieval period, but not in the 100–200 ms sensory window. Such 400–700 ms window after stimulus onset is too late for sensory processes and is mostly considered as the component of sustained parietal contralateral negativity (SPCN), which is indicative of attentional orienting and memory retrieval in the context of change detection task (Tseng et al., 2012b; Hsu et al., 2014). This timing and the attentional nature would be consistent with the larger P3 amplitude reported by Reed et al. (2013) using an orienting task. As such, these results strongly suggest an altered attentional processing during the attentional processing period within the VSTM retrieval stage.

Lastly, it is worth noting that a hand-induced difference in encoding processes is also observed, although its marginal statistical significance prevents us from further exploration into its time windows and particular sites (Figure 3). However, it may be useful to point out that the ERP amplitude is higher in the proximal condition during the encoding period, which possibly suggests a stronger attentional engagement or encoding process. However, this attentional engagement, even if true, seems not to be very helpful, or else we would have observed an enhancement in behavioral performance. In the context of the present study, we have observed a shift of attentional bias toward the right side, but no enhanced performance in the proximal condition over the distal condition.

### The Significance of Complexity in EEG Signals

The notable contribution of this study is the use of MSE as an index for charactering the dynamic changes in EEG signals. Although the neural mechanism of such signal complexity is not yet known, it is assumed that biological systems and their related signals tend to reach an optimal level of complexity that is neither too high nor too low (Costa et al., 2008). For example, entropy measures can also be obtained from heartbeat signals, and in such case atrial fibrillation tend to show higher signal entropy because of the random signals at high frequency that seem more complex. Over multiple time scales, however, high-frequency signals get combined together and eventually show lower MSE than healthy heartbeat at scale 12 or above (Costa et al., 2002). As such, MSE has been suggested as an indicator of “meaningful structural richness” in the form of long-range correlations on multiple temporal (and probably spatial) scales, in the midst of underlying biological, chaotic deterministic dynamics (Costa et al., 2002). In a similar vein, researchers have also suggested complexity as a way of quantifying how the brain codes information within neural signals; therefore, higher signal complexity would be indicative of an information-rich biological system (Deco et al., 2010; Heisz et al., 2012).

In the field of cognitive neuroscience, the concept of “adaptability” has recently been associated with MSE in EEG signals and neural processing. This is based on the view that biological systems need to achieve rapid adaptability in the face of fast environmental changes, which presumably requires integrative multiscale functionality. In the world of cognitive neuroscience, this “environmental change” would be equivalent to the purposely designed task structure of the experiment, and most importantly, the cognitive (and neural) responses that they demand. For example, using a stop-signal task that is designed to induce inhibitory control mechanism, studies have shown that people who are better able to suppress a motor response tend to show higher EEG complexity in MSE analysis. This is true in between-subject studies (Huang et al., 2015), as well as within-subject studies where the same participants’ EEG signals are measured pre- and post-intervention (Liang et al., 2014). In a VSTM study, Wang et al. (2014) also showed that physically active elderly adults had higher EEG signal complexity compared to their sedentary counterparts. Because most of the cognitive tasks (including the ones in our study) involve multiple fast-paced presentations of visual or auditory stimuli on the computer screen, and require an accurate and prompt response from the participants, it is plausible that such temporally and cognitively demanding interaction would require more information capacity and “structural richness” (Costa et al., 2002, 2005; Garrett et al., 2013). In EEG, this structural richness has been hypothesized to be achieved via coherence in neural oscillations and interregional communication (e.g., Buzsáki and Schomburg, 2015), where oscillatory coherence in lower frequencies is crucial for long-range interregional information transfer (Lisman and Buzsáki, 2008; Lisman and Jensen, 2013), which possibly is what high MSE time scales have preserved in our results. If this is true, it would make sense that the MSE effects in cognitive studies tended to focus on higher time scales (Liang et al., 2014; Wang et al., 2014; Huang et al., 2015), whereas resting-state studies that involves no cognitive tasks tended to focus on lower time scales (e.g., Yang et al., 2013). In the context of our observations, proximal and distal conditions also showed a significant difference during the retrieval stage between scales 10 and 25 (Figure 6, top panel). Because high-frequency or random noises tend to get “washed out” at higher time scales, our observation here highlights the possibility that long range, large temporal-scale neurophysiological dynamics may be a key factor underlying the effect of hand proximity. This may hint at a low-frequency and long-range connectivity between parietal and other regions, which is worth investigating for future EEG studies. Nevertheless, the observed distal-proximal differences at higher time scales actually demonstrates the importance of multiscale analyses and highlights the value of MSE analysis.

Finally, although our ERP and MSE analyses both converged on parietal sites to be the loci of hand proximity, MSE analysis of the sensor-based signals actually gave a slightly more accurate localization toward the right hemisphere. This right parietal localization is in line with previous fMRI and brain stimulation studies on VSTM, which suggests a higher involvement of the right parietal cortex in processing visuospatial information in VSTM. Therefore, it is possible that MSE can provide a better approximation of brain regions that is previously not available in the traditional ERP approach. However, because the current results are based on electrode-level findings, precise localization based only on MSE results is not possible and would require further research and validation.

### Theoretical Implications

So far two mechanistic explanations have been proposed to account for the phenomenon and effect of hand proximity on visual processing. There is the bimodal neuron account, which stresses the role of bimodal neurons in the parietal and premotor cortices, whose receptive fields move with the hands (Reed et al., 2006; Tseng et al., 2014). There is also the magnocellular account, which emphasizes on the enhanced processing of magnocellular information in lateral geniculate nucleus due to hand proximity (Gozli et al., 2012; Taylor et al., 2015). Particularly, the magnocellular account can offer a new interpretation to some previous findings. For example, Tseng and Bridgeman (2011) have previously argued that hand proximity may have increased participants’ attentional engagement with the visuospatial stimuli due to the stronger bimodal neuron activities (induced by hand proximity; Reed et al., 2006). On the other hand, the magnocellular account would argue that such effect was perceptual (as opposed to attentional), which was driven by the fact that Tseng and Bridgeman did not control for the luminance level of each stimulus on the display, and therefore some brighter colors were perceived better (and consequently remembered better) than other darker colors, which fits the color-insensitive but luminance-sensitive profile of the magnocellular pathway, and can also account for Tseng and Bridgeman’s results that were previously interpreted as an attentional effect. In the context of the current study, our results seem to suggest that the two competing accounts are not mutually exclusive. This is because our behavioral data surprisingly did not show any near-hand advantage for VSTM performance, which in hindsight may have been due to the similar luminance control that we employed to better control for the varying degrees of brightness in VSTM stimuli in the original Tseng and Bridgeman (2011) study. This lack of behavioral effect due to better luminance control, however, would be consistent and predicted by the magnocellular account (Taylor et al., 2015). Interestingly, despite the lack of enhancement effect in color change detection, hand proximity still biased participants’ attention to the locations near the hands (Figure 2, right panel), which is highly similar to the regional gain patterns observed by Tseng and Bridgeman (2011) and the right bias reported by Le Bigot and Grosjean (2012). Indeed, as previously mentioned, we also observe strong parietal activities induced by hand proximity in both ERP and MSE analyses, which is temporally too late for early visual processing and is more compatible with the bimodal attentional account. These consistent observation of biased attentional shift to the right side (i.e., dominant hand side) has been attributed to the bimodal neurons that respond both to visual and tactile stimuli, which biases one’s attention to the “action space” (i.e., usually where the dominant hand is). Neurophysiological support comes from findings that monkey’s right parietal cortex also shows stronger activities toward their free-moving limbs, and such activity can even transfer to tools held by the hand once the tool has been well-practiced in use (and thus well incorporated into one’s body schema; Graziano and Botvinick, 2002; Reed et al., 2006; Tseng et al., 2012a). Our results are also consistent with this account.

Taken together, our results seem to suggest a dissociable mechanism between altered magnocellular processing and attentional bias near the hands – where the absence of the brightness-driven magnocellular enhancement does not hinder the occurrence of attentional bias toward the dominant hand. That is, the two systems can operate independently, where enhanced magnocellular processing (though absent here due to luminance control) is activated by hand proximity, and such information then gains biased attentional processing in the 400–700 ms time window. If true, this would imply that the attentional and magnocellular accounts may not be mutually exclusive, and such compatibility between the two accounts would explain why both accounts have received ample empirical support in the past decade (Reed et al., 2013; Tseng et al., 2014; Taylor et al., 2015; Thomas, 2015; Thomas and Sunny, 2017a, b). However, this compatibility between the two accounts for now remain a speculation based on the current results, and would need experiments specifically designed to test its plausibility. Nevertheless, the present study demonstrate the utility of MSE analysis on EEG data in the context of hand proximity effects, which opens up many new questions for future investigations. Future studies should look into the biological underpinnings behind the low-frequency and long-range connectivity that is often observed in MSE at hightime scales, as well as the possible spatial selectivity of MSE over ERP approaches, when trying to apply MSE to EEG analysis in the cognitive domain.

## Data Availability Statement

The datasets presented in this article are not readily available because participants did not consent to the sharing of their data. Requests to access the datasets should be directed to tsengphilip@gmail.com.

## Ethics Statement

The studies involving human participants were reviewed and approved by Institutional Review Board of National Cheng Kung University Hospital. The patients/participants provided their written informed consent to participate in this study.

## Author Contributions

Both authors designed the experiment, analyzed the data, and wrote the manuscript.

## Conflict of Interest

The authors declare that the research was conducted in the absence of any commercial or financial relationships that could be construed as a potential conflict of interest.

## Footnotes

1 The algorithm of MSE analysis can be downloaded at http://www.psynetresearch.org/tools.html.
